# Comprehensive Description of an Automated Drug Dispensing System Database

**DOI:** 10.2147/POR.S488210

**Published:** 2025-04-08

**Authors:** Nina L Wittwer, Christoph R Meier, Martin J Recknagel, Samuel Allemann, Cornelia Schneider

**Affiliations:** 1 Department of Pharmaceutical Sciences, University of Basel, Basel, Switzerland; 2 Hospital Pharmacy, University Hospital Basel, Basel, Switzerland; 3 Medifilm AG, Oensingen, Solothurn, Switzerland

**Keywords:** medication blistering, drug therapies, medication changes, drug utilization, polypharmacy

## Abstract

Medifilm is a company that blisters drug therapies recorded by pharmacists in the Medifilm software. The Medifilm dataset collates this information and provides details on drug substances, dosages, pharmacotherapy duration, the sequence of therapies, as well as demographic data on the patients. This article aims to provide an overview of the database, to describe the contents, and to demonstrate possibilities for researchers. The database and the recorded information were described. Furthermore, the data coverage was characterized in terms of the number of available pharmacies, patients, and their drug regimens. The database has been recording data since 2013 and has registered 470,801 blistered therapies for 45,594 patients ordered by 441 pharmacies so far. The longitudinal nature of the database allows researchers to study drug utilization, including medication changes, initiations, and discontinuations over time.

## Introduction

In Switzerland, basic health insurance is mandatory for all individuals.^1^ The Federal Office of Public Health defines what must be covered in the basic health insurance, and all health insurances providing basic health insurance must cover at least this minimal set.^2^,^3^ Basic health insurance covers weekly dispensing systems if a patient has a prescription for it from a medical doctor and takes at least three medications per week. The services can be reimbursed at most once per week.^4^ In Switzerland, most medications need to be prescribed by a medical doctor.^2^ Drugs can then - depending on the canton - either be obtained directly from the prescribing doctor (self-dispensing cantons), or patients can redeem the prescription at a pharmacy of their choice to obtain the drug.^5^

**Figure 1:**
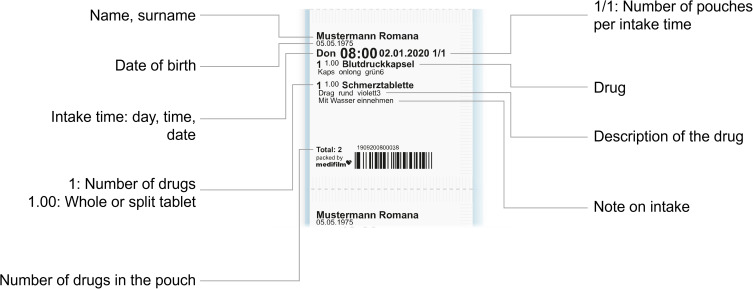
Medifilm^®^ pouch, adapted with permission from Medifilm AG.^7^

**Figure 2:**
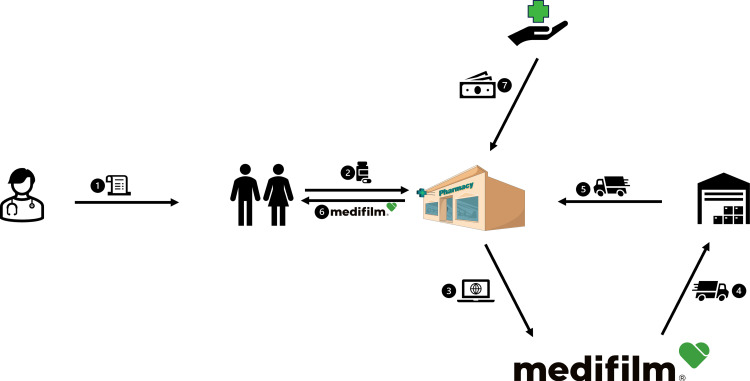
Work flow, adapted with permission from Medifilm AG and Servier Medical Art.^7^,^8^ The prescription issued by the treating doctor (1) authorises a patient to obtain the medication from a pharmacy (2). Pharmacies can order Medifilm^®^ for a patient using an internet-based software (3). After blister packaging through automated packaging machines, the finished Medifilm^®^ is delivered via pharmaceutical logistic partner (4) to the pharmacy (5) and from there to the patient (6). Health insurances reimburse the service if the pharmacy serves patients who have to take at least three different medications per week according to a doctor’s prescription (7).

Automated drug dispensing systems (ADDs), including drug blister packaging, have been demonstrated to reduce errors in drug administration.^6^ These systems are particularly beneficial for populations at higher risk of medication errors, such as patients prescribed multiple medications or those aged 75 years or older, and are therefore a viable option for care homes. By automating the dispensing process, ADDs aim to improve patient safety and support better health outcomes. In Switzerland, medications are repacked and blistered in pharmacies or specialized companies. Since 2007, Medifilm AG has been repackaging the medication of patient-tailored therapies in a continuous soft pouch string. The string (Medifilm^®^) is rolled up per patient and per order and packed for delivery. The service is primarily used for home residents. Each pouch is imprinted with information on the patient, the intake time, the number of medications, medication details, and usage instructions (Figure 1). Pouches can hold solid medications such as tablets or capsules but are not suitable for liquid or semi-solid formulations. Empty pouches with a comment can be added to the pharmacotherapy as a memory aid for the patient or nursing staff, if medications are not suitable for blistering. Pharmacies can order Medifilm^®^ for a patient using an internet-based software (Figure 2). The patient file containing information on therapies and master data is managed by the pharmacies. Therefore, the Medifilm database contains all patient data registered by the pharmacy using the Medifilm software, including demographics, medications, therapies, and ordered blistered medications.

**Figure 3:**
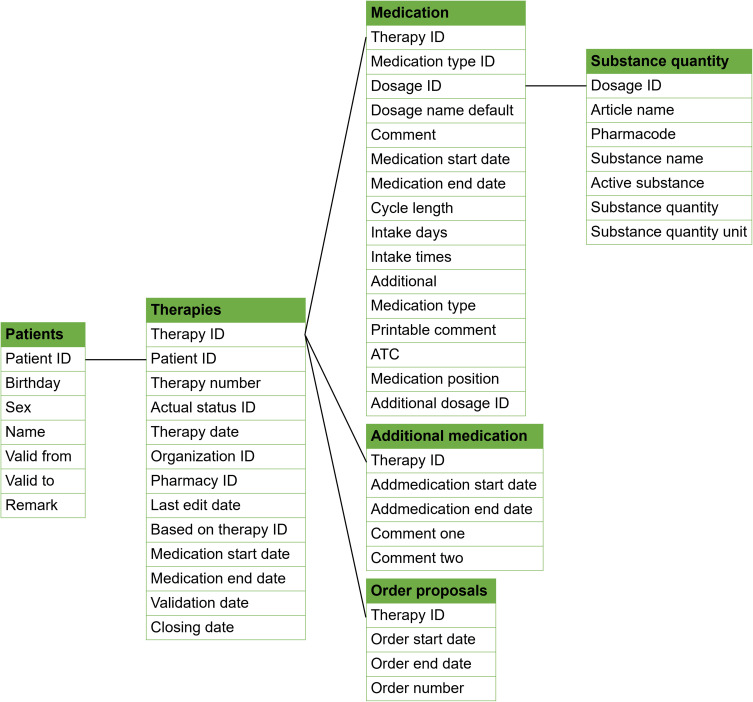
Database architecture.

The Medifilm database records detailed information on patients who have ordered Medifilm^®^, such as information on patient demographics eg, age and sex. In addition, all drugs that can be blistered are added to a patient’s pharmacotherapy list. Any other drug can be selected from the list of known medicines and medicinal products or added as free text. The start and end dates of each pharmacotherapy are also documented, along with each order of the pharmacotherapy as physical blister roll. Information on the medication, dosage, pharmacotherapy duration, and admin time and date are provided. An overview of the main variables and the database architecture are provided in Table 1 and Figure 3. Linkage to other data, like electronic health records or hospital data, is currently not available.

**Table 1:** Summary of Data Tables and Fields

Data Table	Description	Examples of Fields
Patients	Patient demographics	Patient ID, birthday, sex, status of the patient, medical remarks
Therapies	Pharmacotherapies of patients	Therapy ID, status of the therapy, date of therapy creation, date of last edit, base therapy, validation date of pharmacist, therapy end date, pharmacy ID
Order Proposals	Order proposals of therapies	Start and end date of intake, order number
Medication	Medicaments in therapies	ATC, medication intake rhythm, medicament ID, name of medicament, comment for a medication, start and end date of medication, scheme of intake days and quantity, scheme of time and quantity, additional medication not blistered by Medifilm AG, comments printed on pouch
Additional Medication	Additional medication not blistered by Medifilm AG	Comment, start and end date of medication
Substance Quantity	Quantity of substances of a drug	Quantity, Unit, distinction between active ingredient and excipients

## Data Coverage

As of June 2023, the Medifilm database covers 3,582,060 orders. Out of 47,187 patients, 45,594 (96.6%) ordered 470,801 therapies through 441 pharmacies (Table 2). The pharmacies were located in 23 of Switzerland’s 26 cantons (not in Obwalden, Glarus, and Appenzell Innerrhoden) and covered all language regions. The average time between the first and last order of a pharmacotherapy of a patient was 534.7 ± 641.0 days. The average age of patients at the time of their first order was 77.0 ± 17.5 years. On average, each patient received 10.3 ± 13.0 pharmacotherapies, with each pharmacotherapy consisting of an average of 6.8 ± 3.0 drugs. A patient had on average 8.4 ± 4.6 different drugs.

**Table 2:** Medifilm Database Characteristics

Number of	[n]	Percentage [%]
Orders	3,582,060	
Therapies	482,039	
Ordered therapies	470,801	97.7
Pharmacies with therapies	441	
**Number of patients n=47,187**		
Female	27,319	57.9
Male	18,344	38.9
Missing	1524	3.2
**Number of patients with n=47,187**		
Orders	45,594	96.6
**Number of patients with n=45,594**		
Medication changes	39,329	86.3
Age at first order		
12–20 years	295	0.6
21–40 years	2446	5.4
41–60 years	4841	10.6
61–80 years	11,795	25.9
81–100 years	26,037	57.1
>100 years	180	0.4
1–5 therapies	21,694	47.6
6–10 therapies	9358	20.5
11–15 therapies	5200	11.4
16–20 therapies	3129	6.9
21–40 therapies	4734	10.4
>40 therapies	1479	3.2
1–5 drugs	12,585	27.6
6–10 drugs	20,890	45.8
11–15 drugs	8869	19.5
16–20 drugs	2395	5.3
>20 drugs	855	1.9
1–5 drugs at the same time	14,889	32.7
6–10 drugs at the same time	24,221	53.1
11–15 drugs at the same time	6081	13.3
16–20 drugs at the same time	380	0.8
>20 drugs at the same time	23	0.1

## Discussion

To ensure patient safety, it is crucial that the recorded data - relevant to the pharmacotherapy that is blistered - is of high quality. Pharmacists or pharmaceutical technicians enter patient data into Medifilm software. Various checks are performed to ensure the quality of the recorded data and to detect input errors. These include checking for maximum values, avoiding duplicate rows, displaying additional information such as crushability or suspendability, and displaying the drug picture. When validating the pharmacotherapy, additional information such as alternative preparations or drug interaction checks are displayed. All data is validated by an authorized pharmacist. Authorization is granted following an application including verification at the MedReg register of medical professionals.^9^ It is also possible to compare a patient’s new pharmacotherapy with an old pharmacotherapy.

A strength of the Medifilm database is that it provides detailed information on patient medication, including pharmacotherapy changes such as dosage adjustments, medication initiation, and discontinuation, which are entered by health care professionals. Furthermore, the Medifilm database offers longitudinal data, currently spanning a 10-year period. Compared to other Swiss claims databases, drug information is more frequently updated, providing a more comprehensive picture of a patient’s pharmacotherapy. The patient population in the Medifilm database consists predominantly of elderly individuals with polypharmacy, providing an opportunity to study a multimorbid population.

The quality of the data depends on the accuracy and completeness of information that pharmacies record in the Medifilm software. Data that is not recorded by pharmacies is not captured in the database. The quality of data varies across pharmacies and patients. For example, non-blistered medications, such as liquid medications, patches, suppositories, dermatological preparations, granules, and effervescent tablets might be added as free text in the record of one patient, but not in another. Additionally, the quality of free text data varies. Medifilms^®^ are usually ordered for multimorbid patients, and thus the database is not representative for the general population.

While Medifilm^®^ is a tool designed to support medication adherence, it cannot guarantee that all prescribed medications will be taken.

The Medifilm database has the potential to study medication use and medication changes in patients with polypharmacy and has previously been used to analyze the utilization of splitting tablets.^10^

## Data Availability

The datasets generated and/or analyzed during the current study are not publicly available due to confidentiality requirements issued by Medifilm AG. Analysis codes and datasets can be made available by the corresponding author (s.allemann@unibas.ch) upon reasonable request and with permission of Medifilm AG.

## Data Resource Access

More information about the Medifilm database can be found at https://www.medifilm.ch/de/ and data access can be requested from Medifilm AG (info@medifilm.ch).

## References

[1] Biller-Andorno N, Zeltner T. Individual responsibility and community solidarity — the Swiss Health Care System. *N Engl J Med*. 2015;373(23):2193–2197. doi:10.1056/NEJMp1508256 26630139

[2] Federal Office of Public Health (FOPH). List of Specialties (SL); 2023. Available from: http://www.spezialitätenliste.ch/. Accessed April 7, 2022.

[3] Federal Office of Public Health (FOPH). Analysis List (AL); 2024. Available from: https://www.bag.admin.ch/bag/de/home/versicherungen/krankenversicherung/krankenversicherung-leistungen-tarife/Analysenliste.html. Accessed December 28, 2023.

[4] pharmaSuisse, santésuisse, curafutura. Tarifstruktur-Vertrag LOA IV/1; 2016. Available from: https://pharmasuisse.org/system/files/media/documents/2023-10/Tarifstruktur-Vertrag-LOA-IV-1.pdf. Accessed 26 January 2024.

[5] Arslan S. 21.3881 | selbstmedikation Arzneimittel. Wo stehen wir heute? | amtliches Bulletin | das Schweizer Parlament; 2021. Available from: https://www.parlament.ch/de/ratsbetrieb/amtliches-bulletin/amtliches-bulletin-die-verhandlungen?SubjectId=57767. Accessed March 26, 2024.

[6] Hänninen K, Ahtiainen HK, Suvikas-Peltonen EM, Tötterman AM. Automated unit dose dispensing systems producing individually packaged and labelled drugs for inpatients: a systematic review. *Eur J Hosp Pharm*. 2023;30:127–135. doi:10.1136/ejhpharm-2021-003002 34795001 PMC10176995

[7] Medifilm AG. medifilm.ch. Available from: https://www.medifilm.ch/de/. Accessed March 21, 2024.

[8] Servier. Servier Medical Art. Available from: https://smart.servier.com. Accessed February 23, 2023.

[9] Federal Office of Public Health (FOPH). MedReg register of medical professions. Available from: https://www.bag.admin.ch/bag/en/home/berufe-im-gesundheitswesen/medizinalberufe/medizinalberuferegister-medreg.html. Accessed March 20, 2024.

[10] Allemann SS, Bornand D, Hug B, Hersberger KE, Arnet I. Issues around the prescription of half tablets in Northern Switzerland: the irrational case of Quetiapine. *Biomed Res Int*. 2015;2015:602021. doi:10.1155/2015/602021 26539514 PMC4619813

[11] Fedlex. Federal Act on Data Protection (FADP); 2019. Available from: https://www.fedlex.admin.ch/eli/cc/1993/1945_1945_1945/en. Accessed December 24, 2021.

